# Declining Age-Specific Seroprevalence and Seroconversion Rates in *Plasmodium falciparum* from 2009 to 2018 Documents Progress toward Malaria Elimination in Southern Zambia

**DOI:** 10.4269/ajtmh.22-0401

**Published:** 2023-05-01

**Authors:** Ben Katowa, Harry Hamapumbu, Philip E. Thuma, Sophie Bérubé, Amy Wesolowski, William J. Moss, Tamaki Kobayashi, for the Southern and Central Africa International Centers of Excellence for Malaria Research

**Affiliations:** ^1^ Macha Research Trust, Choma, Zambia;; ^2^ Department of Epidemiology, Johns Hopkins Bloomberg School of Public Health, Baltimore, Maryland;; ^3^ W. Harry Feinstone Department of Molecular Microbiology and Immunology, Johns Hopkins Bloomberg School of Public Health, Baltimore, Maryland

## Abstract

Obtaining accurate malaria surveillance data is challenging in low-transmission settings because large sample sizes are required to estimate incidence and prevalence precisely. Serology is an additional tool to document progress toward malaria elimination. An enzyme immunoassay to *Plasmodium falciparum* lysate was used to estimate age-specific seroprevalence among residents of southern Zambia, where malaria transmission has declined to pre-elimination levels during the past two decades. Plasma was eluted from 3,362 dried blood spots collected during five cross-sectional surveys conducted between 2009 and 2012, and again in 2018. Annual seroconversion rates (SCRs), an estimate of the force of infection, were calculated using a reversible catalytic model. The SCR decreased by two thirds from a level of approximately 0.15/year in 2009 and 2010 to approximately 0.05/year in 2011 and 2012, and then decreased 5-fold to 0.01/year by 2018, demonstrating the utility of serology in documenting progress toward elimination.

Malaria remains a threat to global health, with an estimated 247 million cases and 619,000 deaths attributed to malaria in 2021.^1^ The WHO’s Global Technical Strategy for Malaria 2016–2030 identified surveillance as a core intervention tool for malaria control and elimination.^2^ The 2021 update of the technical strategy further emphasized the importance and challenges of collecting high-quality malaria surveillance data for decision making.^3^ As malaria transmission declines, obtaining high-quality malaria surveillance data is increasingly challenging because large sample sizes are required to estimate incidence and prevalence precisely.

Serological surveillance may offer an additional tool to estimate malaria transmission intensity and document progress toward malaria elimination in low-transmission settings.^4^ A study^5^ conducted in Senegal between 2000 and 2012 measured antibodies against *Plasmodium falciparum* schizont crude extract, and annual seroconversion rates (SCRs)—the rate at which seronegative individuals become seropositive—were calculated. A strong, positive correlation was observed between SCRs and entomological inoculation rates, demonstrating the utility of serological data in assessing malaria transmission intensity in low-transmission settings.^6^

Zambia has set ambitious goals for malaria elimination, originally by 2021.^7^ Southern Province has the lowest parasite prevalence and malaria incidence in the country, and thus is closest to achieving elimination.^8^ Parasite prevalence by microscopy among children younger than 5 years declined from 5.7% in 2010 to 0% in 2018 according to the Malaria Indicator Survey (MIS).^8^^,^^9^ In the 2021 MIS, parasite prevalence for Southern Province by microscopy was not available, but was 3.3% by rapid diagnostic test (RDT).^10^ The inability to detect parasitemia in Southern Province is, however, not an indication that malaria elimination was achieved, but rather the insensitivity of the survey methods to detect both low parasite prevalence and low levels of parasitemia in infected individuals by microscopy or RDT. We conducted serial cross-sectional serosurveys over a broad age range, including adults, to demonstrate that serosurveillance can provide estimates of both declining transmission and waning antibody levels to whole parasite lysate in the catchment area of Macha Hospital in Southern Province, Zambia.

The catchment area of Macha Hospital is located approximately 70 km north of the provincial capital of Choma and is mainly inhabited by subsistence farmers. The area is characterized by Miombo woodland, with a rainy season that lasts from early December through the end of March followed by a cool, dry season from April to July and a hot, dry season from August to November.^11^ Malaria transmission is mainly restricted to the rainy season. The major malaria vector is *Anopheles arabiensis*,^11^ with secondary vectors such as *Anopheles squamosus* also contributing to transmission.^12^ Long-lasting insecticide-treated nets are the main vector control method, although targeted indoor residual spraying is deployed occasionally. After a series of malaria control interventions, pediatric admissions resulting from malaria at Macha Hospital declined more than 90% in 2020 compared with 2003.^13^

Between 2009 and 2012, 533 households in the catchment area of Macha Hospital in Choma District, Southern Province, Zambia, participated in serial cross-sectional, community-based surveys conducted every other month (January, March, May, July, September, and November). Households were selected randomly without replacement, and thus households and individuals participated only once. All household residents were eligible for inclusion. Data were aggregated by calendar year as the unit of analysis. The sampling frame was based on satellite imagery as described previously.^14^ A Quickbird™ satellite image from DigitalGlobe Services, Inc. (Denver, CO) was imported into ArcGIS 9.2 (Redlands, CA), and locations of households were identified and enumerated manually. In 2018, an additional 90 previously unselected households from the study area were enrolled in April, May, and June using the same sampling frame. A total of 3,362 individuals participated across all surveys (2,947 participants between 2009 and 2012, and 415 participants in 2018). Informed consent was obtained from all participants, and guardians provided permission for children younger than 16 years. Parasite prevalence was assessed using *P. falciparum* histidine-rich protein 2 (PfHRP2)–based RDT (SD Bioline Malaria Ag P.f, Standard Diagnostics Inc., Gyeonggi-do, Republic of Korea), and blood samples were collected on Whatman 903 protein saver cards (GE Healthcare, Cardiff, UK). Blood samples were dried overnight, packaged individually with desiccant, and stored at −20°C as dried blood spots.

IgG antibodies to *P. falciparum* were measured using an enzyme immunoassay (EIA) to asexual parasite lysate of *P. falciparum* NF54. Plasma was extracted by soaking the dried blood spots in 5% skim milk and phosphate-buffered saline containing 0.05% Tween 20 for 1 hour at room temperature. IgG antibody levels to whole *P. falciparum* lysate were measured at 405 nm and expressed as the optical density (OD).^15^ A threshold OD value of 0.57 to determine seropositivity was established previously based on the mean OD value plus 3 SDs using filter paper spotted with serum from 10 individuals from the United States who reported no previous travel to *P. falciparum*–endemic areas.^15^ Negative and positive control sera were included in each plate to adjust for plate-to-plate variation.

A previously reported reversible catalytic model was fitted to the age-specific seroprevalence data using Stata version 16 (StataCorp LP, College Station, TX) to estimate annual SCRs.^5^ SCRs provide a measure of the force of infection and the rate susceptible individuals acquire infection; and declining SCRs can be interpreted as evidence of a decreasing force of infection.^16^

Demographic characteristics of the study participants and parasite prevalence by PfHRP2-based RDT by year of enrollment are summarized in Table 1. The distributions by age and gender were comparable across the study period, with the median age ranging from 13 to 14 years and the proportion of males ranging from 44% to 48%. Parasite prevalence by PfHRP2-based RDT declined after 2009, and no participant was positive according to PfHRP2-based RDT in 2018. Observed and predicted age-specific seroprevalence curves were plotted for 2009 to 2012 and 2018 (Figure 1A–E). To illustrate the decline in seropositivity better across all age groups, plots for 2009, 2012, and 2018 were overlain (Figure 1F). Plots from 2009 and 2012 have two phases, a linear phase followed by a plateau phase, and for both phases the proportion seropositive was less in 2012 than 2009. In 2018, the two phases were not well defined, but the proportion seropositive was less. The SCR decreased sharply, by two thirds, from a level of approximately 0.15/year in 2009 and 2010 to ∼0.05/year in 2011, and 2012 and then decreased 5-fold to 0.01/year by 2018 (Table 2^17^^–^^19^). The parasite prevalence by microscopy for children younger than 5 years in Southern Province as measured in the Zambian MIS is included in Table 2 for the years data are available.^8^^,^^9^^,^^17^^–^^19^

**Table 1:** Participant characteristics

Characteristic	Year				
	2009	2010	2011	2012	2018
No. of participants	675	864	736	672	415
Age, years; median (IQR)	14 (6.4–32)	14 (6.0–33)	14 (5.9–36)	13 (5.0–31)	14 (6.0–34)
<5 years, %	20	20	22	23	19
≥ 30 years, %	28	28	33	28	28
Prevalence by RDT, *n* (%, 95% CI)	10 (1.5, 0.71–2.7)	2 (0.23, 0.03–0.83)	3 (0.41, 0.08–1.2)	1 (0.15, 0.004–0.82)	0 (0, 0–0.88*)

IQR = interquartile range; RDT = rapid diagnostic test.

* One-sided, 97.5% CI.

Number of participants, median age, and parasite prevalence by *Plasmodium falciparum* histidine-rich protein 2–based RDT by year of enrollment. Serial cross-sectional collections occurred from 2009 to 2012, with additional collections in 2018. Samples were collected once and were never repeated for the same individual.

**Figure 1.**
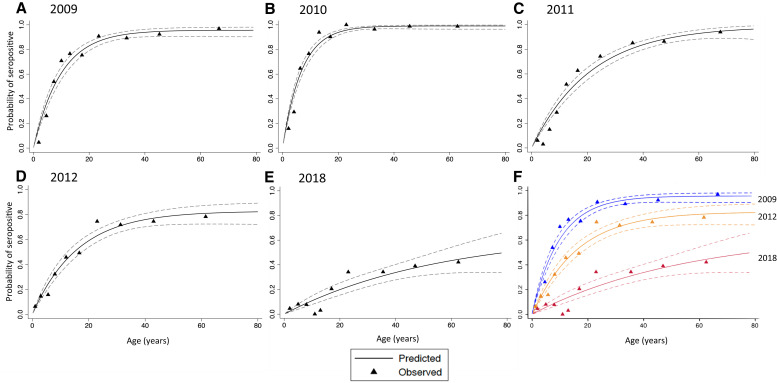
Observed and predicted seroprevalence by year. The proportion seropositive as predicted by the reversible catalytic model (solid lines) and observed data (solid triangles) with 95% CIs intervals (dashed lines) from (**A**) 2009, (**B**) 2010, (**C**) 2011, (**D**) 2012, and (**E**) 2018. (**F**) Plots from 2009, 2012, and 2018 are overlain.

**Table 2:** Annual SCRs across all ages, with seropositivity stratified by two age groups (<5 and ≥30 years), and parasite prevalence by microscopy for 2008 to 2015 and 2018.

Year	SCR (95% CI)	Seropositivity by age group, years; % (95% CI)		Parasite prevalence by microscopy in Southern Province (Zambia MIS)
		<5	≥30	
2008	–	–	–	7.9^9^
2009	0.16 (0.14-0.19)	26 (19–35)	98 (95–100)	N/A
2010	0.15 (0.13-0.17)	20 (14–27)	98 (95–99)	5.7^17^
2011	0.046 (0.040-0.054)	4.7 (1.9–9.4)	83 (78–88)	N/A
2012	0.055 (0.045-0.068)	13 (8.3–20)	74 (68–80)	8.4^18^
2015	–	–	–	0.6^19^
2018	0.012 (0.009-0.018)	2.5 (0.3–8.8)	37 (28–47)	0^8^

MIS = Malaria Indicator Survey; N/A = not applicable; SCR = seroconversion rate.

The annual SCR was modeled using participants’ serostatus from each year (characteristics of participants are described in Table 1). Seropositivity by age group is a proportion of seropositivity for those younger than 5 years and those 30 years and older. Parasite prevalence by microscopy among children younger than 5 years was reported in the Zambia National MIS conducted by the Zambian Ministry of Health.

Reasons for the sharp decline in SCRs are not entirely clear, but likely are a consequence of malaria control interventions,^13^ including insecticide-treated bed nets and reactive test-and-treat strategies, as well as a drought that may have reduced *Anopheles funestus* abundance dramatically.^11^ The decline in seroprevalence among participants 30 years and older is consistent with waning antibody levels to whole parasite lysate. Serology thus combines in a single measure quantitative estimates of the force of infection that can be used to monitor changes over time, as well as potential increased susceptibility in adults, recognizing that antibodies to whole parasite lysate are not a correlate of protective immunity.

Many serological studies of malaria used a selected set of *P. falciparum*–specific antigens such as merozoite surface protein 1 and apical membrane antigen 1.^4^ We used an EIA with asexual-stage parasite lysate as the antigens to measure a broad range of *P. falciparum*–specific IgG antibodies. Whole parasite lysate was prepared in-house, and the EIA was a relatively low-cost and easy-to-implement assay to measure changes in age-specific seroprevalence in this low-transmission setting. A serological assay that measures antibodies to a broad range of parasite antigens may be a sensitive measure of exposure to *P. falciparum*. A serological survey conducted in the same study area in southern Zambia from 2013 to 2015 using a protein microarray revealed the breadth and depth of IgG antibody responses to 500 different *P. falciparum* peptides,^20^ but the overall age-specific seroprevalence curve was similar to that generated with whole parasite lysate. For tracking changes in age-specific seroprevalence to *P. falciparum* in low-transmission settings, an EIA using whole parasite lysate may be sufficient.

As malaria transmission declines, commonly used diagnostic tests such as RDTs or microscopy fail to detect prevalent infections. The use of polymerase chain reaction testing becomes cost prohibitive because of the large sample size requirements for precision. Unlike polymerase chain reaction testing, which detects active infections, serosurveys can detect past infections, alleviating the issue of sample size. Serosurveys using an EIA described in this article could be performed readily by national malaria control programs, and information from serial cross-sectional serosurveys can provide an additional quantitative measure of declining malaria transmission intensity in pre-elimination settings.
